# Impact of bubble size in a rat model of cerebral air microembolization

**DOI:** 10.1186/1749-8090-8-198

**Published:** 2013-10-18

**Authors:** Martin Juenemann, Mesut Yeniguen, Nadine Schleicher, Johannes Blumenstein, Max Nedelmann, Marlene Tschernatsch, Georg Bachmann, Manfred Kaps, Petr Urbanek, Markus Schoenburg, Tibo Gerriets

**Affiliations:** 1 Department of Neurology, Justus-Liebig-University Giessen, Giessen, Germany; 2 Heart & Brain Research Group, Justus-Liebig-University Giessen and Kerckhoff Clinic, Benekestraße 2-8, Bad Nauheim 61231, Germany; 3 Department of Radiology, Kerckhoff Clinic, Bad Nauheim, Germany; 4 Department of Cardiac Surgery, Kerckhoff Clinic, Bad Nauheim, Germany; 5 Department of Neurology, Buergerhospital Friedberg, Friedberg, Germany

**Keywords:** Animal experimentation, Cerebral infarction, Cardiopulmonary bypass, Cardiac surgical procedures, Gas embolism

## Abstract

**Background:**

Cerebral air microembolization (CAM) is a frequent side effect of diagnostic or therapeutic interventions. Besides reduction of the amount of bubbles, filter systems in the clinical setting may also lead to a dispersion of large gas bubbles and therefore to an increase of the gas–liquid-endothelium interface. We evaluated the production and application of different strictly defined bubble diameters in a rat model of CAM and assessed functional outcome and infarct volumes in relation to the bubble diameter.

**Methods:**

Gas emboli of defined number and diameter were injected into the carotid artery of rats. Group I (n = 7) received 1800 air bubbles with a diameter of 45 μm, group II (n = 7) 40 bubbles of 160 μm, controls (n = 6) saline without gas bubbles; group I and II yielded the same total injection volume of air with 86 nl. Functional outcome was assessed at baseline, after 4 h and 24 h following cerebral MR imaging and infarct size calculation.

**Results:**

Computer-aided evaluation of bubble diameters showed high constancy (group I: 45.83 μm ± 2.79; group II: 159 μm ± 1.26). Animals in group I and II suffered cerebral ischemia and clinical deterioration without significant difference. Infarct sizes did not differ significantly between the two groups (*p* = 0.931 *u*-test).

**Conclusions:**

We present further development of a new method, which allows reliable and controlled CAM with different bubble diameters, producing neurological deficits due to unilateral cerebral damage. Our findings could not display a strong dependency of stroke frequency and severity on bubble diameter.

## Background

Cerebral air microembolization (CAM) as an iatrogenic event represents a well-known complication of various invasive medical interventions. In the fields of open-heart surgery, neurosurgery, and diagnostic or therapeutic neuroradiology, CAM contributes to increased periprocedural morbidity and mortality, warranting the development of preventive strategies [1-3].

Severe CAM, i.e. large volume cerebral air embolization, is a rare condition that results in severe brain damage. According to clinical observations and animal studies, fatality caused by the infusion of large gas volumes seems to be undisputable [4,5]. To date, the consequences of CAM with small amounts of embolized air are still unknown. Frequently seen in cardiopulmonary bypass (CPB) assisted surgery or angiography, CAM can lead to persistent focal neurological and neuropsychological deficits, often without the display of manifest cerebral infarction on MRI [2,6]. Recent clinical trials suggest that the diminution of CAM can improve neuropsychological outcomes within the first three months post intervention [2]. Pathophysiological background, thresholds for emboli load, and other determinants of clinical outcome after embolization are largely unknown.

Mechanisms of CAM-induced tissue damage exceed mere mechanical consequences such as compression against the endothelial capillary wall and obstruction of blood flow by a lodging bubble [7]: Constituting a foreign substance, air bubbles provoke clotting activation [8-11], inflammatory response with neutrophil aggregation and complement activation [12,13] with the production of radical species leading, amongst others, to an increased vascular permeability and triggering a complex immunological and ischemic cascade of tissue injury. These pathomechanisms are initiated on the surface of the bubble with the gas–liquid-endothelium interface as a driving force [7].

The use of filter systems during CPB-assisted surgery can reduce the amount of gaseous cerebral emboli, but it seems conceivable that the mesh-like structure of these filters may also cause a dispersion of large gas bubbles into numerous smaller particles. This would substantially increase the total bubble surface and, therefore, intensify the potentially hazardous gas–liquid-endothelium contact in turn. Not least because of the absence of an appropriate animal model and the inability to control and monitor the diameter of the bubbles, knowledge of cerebral air embolism is sparse and little is known about the influence of bubble size [14].

Based on the hypothesis that bubble size has an impact on the neurological outcome and the occurrence of cerebral ischemia following CAM, we aimed to prove feasibility and reliability of production as well as application of gaseous bubbles with different defined bubble diameters by means of a recently developed custom-made bubble generator [15]. Furthermore, we evaluated embolization effects with regard to the functional neurological outcome, frequency and volume of cerebral ischemia in relation to bubble diameter.

## Methods

### Production and application of microbubbles

**Figure 1:**
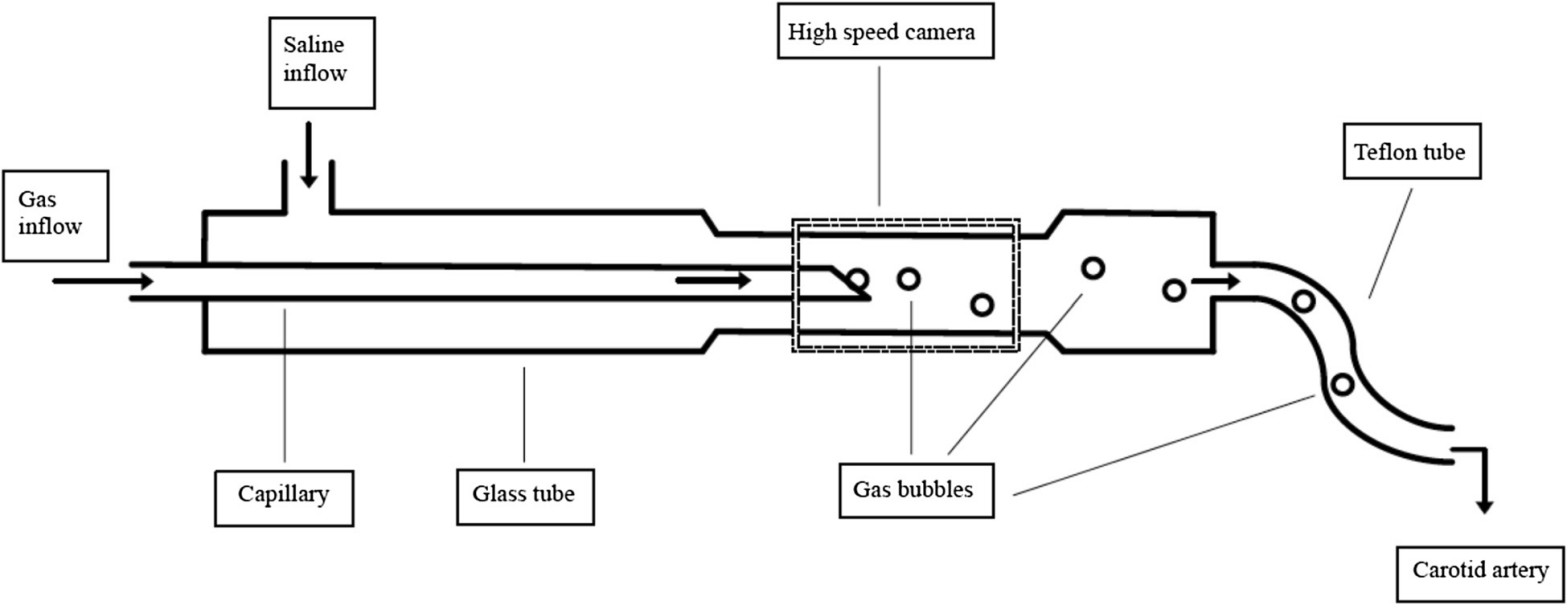
**Schematic diagram of the custom-made bubble generator.** Controlled alteration of air bubble number and size can be obtained by the variation of airflow, saline flow, diameter, and position of the capillary within the outer tube. Automatic recording and determination of bubble number and size is performed using a high-speed optical image capturing system connected to a computer with custom-made software.

For the production and application of air microbubbles, we used a custom-made device, as previously established by our group [15]: the device consists of a customized glass capillary with a diameter of 15-40 μm, positioned in a larger glass tube with a diameter of 450 μm (Rincaps, Hirschmann Laborgeraete GmbH) and a slight taper on one side (Figure 1). The space between these two tubes was jetted with saline at a constant flow of 20 ml/h driven by an infusion pump (MC Medizintechnik GmbH, Alzenau). Simultaneously, air at a precisely adjustable pressure, controlled by a regulator (Watson Smith Ltd., Leeds, England), was piped through the inner capillary, requiring pressure from 0.5 to 1.5 bar to vary the number of produced bubbles.

The number and size of air bubbles were carefully adjusted at a constant rate before the device was connected to the carotid artery of the rat. During this process, a hydrostatic pressure was obtained within the system simulating the physiological mean arterial blood pressure of each animal under anesthesia. After adjustment, a glass valve between the Teflon tubing and the glass tube was opened and saline with air bubbles was allowed to flow into the carotid artery of the rat, joining the bloodstream.

### Quantification of the number and size of air microbubbles

The bubble formation was recorded by a high-speed optical image capturing system (Camera A602f-2, Basler, Switzerland) focusing on the tapering (Figure 1) and connected to a personal computer (MacBook Pro, Mac OS X, version 10.5.1). A custom-made software allowed real-time recording and analysis of the number and size of the air bubbles flowing into the carotid artery of the animal.

### Animal preparation

All procedures were carried out in accordance with our institutional guidelines and the German animal protection legislation and were approved by the regional ethics committee (Regierungspraesidium Darmstadt; AZ B2/203). Twenty male Wistar Unilever rats (HsdCpb:WU; Harlan Winkelmann, Borchen, Germany) were anesthetized with 5% isoflurane in an enclosed chamber. During surgery, anesthesia was maintained with 2-3% isoflurane delivered in air at 0.5 l/min through a closely fitting facemask under sustained spontaneous breathing. Analgesia was achieved by subcutaneous injection of Carprofen 5 mg/kg (Rimadyl, Pfizer, Germany) 30 min prior to surgery. During these procedures, the body temperature of the rat was continuously monitored with a rectal probe and maintained at 36.5-37°C with a thermostatically controlled heating pad.

The right common carotid (CCA), internal carotid (ICA), and external carotid artery (ECA) were exposed by a midline incision of the neck. After isolation of the ECA, the superior thyroid and occipital arteries were cauterized. Then the distal portion of the ECA was ligated with a 5–0 suture and intersected to create an ECA stump with a length of approximately 5 mm. Furthermore, the pterygopalatine branch of the ICA was ligated with a 5–0 suture.

### Application of air bubbles to cerebral circulation

The Teflon-50 tubing (Figure 1) was deaerated, connected with the air bubble generator, and then inserted into the ECA stump through an arteriotomy. To ensure the free movement of the air bubbles with the current of the physiological blood stream from the CCA to the ICA, the tip of the tubing was placed close to the carotid bifurcation under visual control. Unintended inlet of air was avoided by fixation of the tubing within the ECA stump through ligature and controlled optically via the surgical microscope during the whole injection procedure.

To minimize total injection volume, we chose the lowest infusion speed carrying the air bubbles appropriately: the infusion was performed within approximately 2 minutes at a speed of 20 ml/h, equivalent to an infusion volume of approximately 0.65 ml saline per animal. A PE-50-catheter was inserted into the tail artery from 30 minutes before until 30 minutes after carotid artery infusion in order to monitor the arterial blood pressure and obtain blood samples for arterial blood gas analysis. After air infusion, the ECA was ligated, the tubing removed, and the wounds closed carefully. Then the animals were allowed to recover from anesthesia.

### Functional testing

Functional testing was performed by investigators blinded to group assignment at baseline, after 4 h, and after 24 h using a Neuroscore, as previously described by Nedelmann et al., and the Rotarod test: The Neuroscore assesses dimensions of motor, coordinative, and sensory function by testing contralateral forelimb flexion, hemianopia, sensory to left side touch, instability to lateral push from the right, and coordination when walking on the ground. For each item, up to 10 points can be allocated. The Neuroscore can range from 0 (no deficit) to 90 (very severe neurological deficit) [16].

During Rotarod testing, the wheel was continuously accelerated from 0 to 30 rpm within one minute. The maximum speed tolerated by the rats without falling off or spinning around was documented [17].

### MR imaging

MR imaging was carried out 24 hours after intervention. Animals were fixed in a body restrainer with a tooth bar and a cone-shaped head holder and placed in an MRI tomograph (Bruker PharmaScan 7.0 T, 16 cm, Ettlingen, Germany), which operates at 300.51 MHz for ^1^H-imaging and is equipped with a 300 mT/m self-shielding gradient system.

The respiratory rate (50-70/min) was monitored with a pressure probe placed between the restrainer and the animal’s thorax. Anesthesia was maintained with isoflurane delivered in air at 0.5 L/min. The isoflurane concentration was varied between 2.0% and 3.0% to keep the respiratory rate between 35 and 45/min. The temperature was monitored using a rectal probe and maintained at 37°C by a thermostatically regulated water flow system throughout the imaging protocol.

A Carr-Purcell-Meiboom-Gill spin echo imaging sequence was used to map lesion and hemisphere volumes. Six contiguous coronal slices with a thickness of 2 mm (gap of 0 mm) were acquired (Field of view 37 × 37 mm, matrix 512 × 256, repetition time 3833.5 msec, 12 echoes: echo time 18–216 msec (Δ echo time 18 msec), acquisition time 16.25 minutes, number of excitations 1).

The T2-relaxation time was measured in regions of interest within the center of the ischemic area on all slices displaying ischemic lesions and a corresponding position on the contralateral hemisphere. The difference in T2RTs between the ischemic and unaffected hemispheres was calculated.

Computer aided planimetric assessment of ischemic lesion volumes and hemispheric volumes were performed by two blinded investigators experienced in experimental stroke MRI. The ipsilateral and contralateral hemispheric volume and lesion volume on T2WI were determined using the Image J 1.25 s image analysis software (National Institutes of Health, USA). The edges of the hemispheres were traced manually on each slice using neuroanatomic landmarks. Hyperintense ischemic lesions were traced manually in a similar fashion. The areas were then summed and multiplied by the slice thickness to calculate volumes. Lesion volumes were calculated with and without edema correction and expressed as a percentage of the hemispheric volume as described previously [18]:

$$\%\mathrm{HLVuc}=\mathrm{LV}/\left(\left(\mathrm{HVc}+\mathrm{HVi}\right)/2\right)*100)$$

$$\begin{array}{ll} \%\mathrm{HLVec}= & \left(\mathrm{HVc}2+\mathrm{LV}*\left(\mathrm{HVc}+\mathrm{HVi}\right)–\mathrm{HVi}2\right)/\\ & \left(\mathrm{HVc}*\left(\mathrm{HVc}+\mathrm{HVi}\right)\right)*100 \end{array}$$

(%HLVuc = percent hemispheric lesion volume – not corrected for edema; %HLVec = percent hemispheric lesion volume – corrected for edema; HVc = volume of the contralateral hemisphere; HVi = volume of the ipsilateral hemisphere; LV = lesion volume)

### Experimental protocol

Twenty rats (body weight 300-347 g) were randomized into 3 groups: the animals in group I (n = 7) received 1800 air bubbles with a low target diameter of 45 μm. Group II (n = 7) received 40 air bubbles with a higher target diameter of 160 μm, yielding equal total volume of administered air per rat of 86 nl in both groups. The animals in Group III (n = 6) served as sham-operated controls with the infusion of an identical volume of saline without application of air bubbles. Functional testing was performed at baseline, at 4 h, and at 24 h after intervention, followed by MRI examination.

### Statistical analysis

The Kolmogorov–Smirnov Test was used to test for normal distribution of data; homogeneity of variance was tested by Levene-Test. Group differences were tested by non-parametric Kruskal-Wallis Test. Then the individual groups were subjected to subsequent post-hoc analysis with Mann-Witney-*U* Test. The p-value *p* < 0.05 was considered statistically significant. The data are presented as mean ± standard deviation.

## Results

Four animals had to be excluded from analysis: one animal in group I received bubbles with a mean diameter much higher than the target size of 45 μm due to technical problems in adjusting the bubble size. During bubble injection of an animal in group II, flow problems occurred and bubbles conglomerated at the tip. Another rat in group II died 5 hours after the intervention. A fourth animal showed severe hemodynamic instability as well as apneas during saline application. The remaining sixteen animals (group I n = 6, group II n = 5, group III n = 5) survived and completed the study protocol.

### Reliability of air bubble generation

For the present experiment, we selected two groups with target air bubble diameters of 45 and 160 μm compared to a sham-operated control group. The actual bubble sizes, as determined by the computer-aided camera system, were 45.83 μm ± 2.79 (range 42-49 μm) and 159 μm ± 1.26 (range 157-160 μm). The effective bubble count injected into the carotid artery averaged out at 1808.5 ± 11.04 in group I and 40.17 ± 0.41 in group II.

### Functional outcomes

In the sham-infusion group, no pathological findings in Neuroscore performance were detectable. Animals in groups I and II showed clinical deterioration after bubble infusion as determined by the Neuroscore: In group I, the mean neurological score was 15 ± 11.83 after 4 h and 10 ± 5.47 after 24 h, and in group II, it was 19.17 ± 11.14 after 4 h and 19 ± 10.24 after 24 h. Compared to the control group (III), these findings appeared to be statistically significant. However, between the two bubble sizes of 45 and 160 μm, differences after 4 h (*p =* 0.931) and 24 h (*p =* 0.177) did not reach statistical significance.

In animals of all groups, the Rotarod performance worsened slightly after injection. At baseline, the mean maximum speed tolerated by the animals of group I was 23 ± 4.52 rpm; at 4 h and 24 h after intervention, test performance decreased to 21 ± 7.35 rpm for both time points. Animals in group II tolerated a mean speed of 22 ± 3.10 rpm at baseline (*p =* 0.664), 19 ± 11.01 rpm after 4 h (*p =* 0.792), and 20.4 ± 10.90 rpm after 24 h (*p* = 0.931). Between these two groups, no statistical significance could be detected.

### Cerebral infarction on MRI

**Figure 2:**
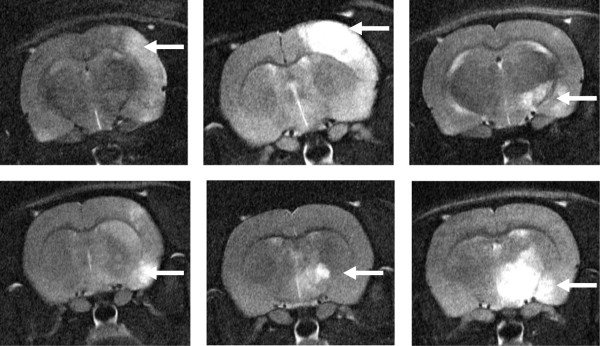
**Cerebral MRI.** Representative examples of T2-weighted MRI 24 h after embolization of small (1st row) or large (2nd row) bubbles. Note the arrows indicating cortical and subcortical ischemic lesions displayed in white.

**Figure 3:**
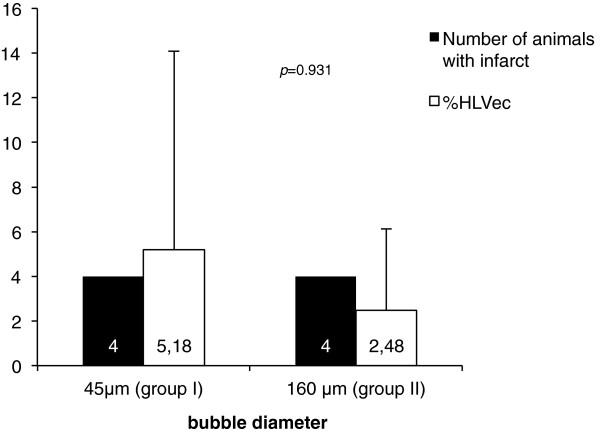
**Number and size of cerebral infarction.** Four animals in each group suffered cerebral infarction detectable on MRI. Infarct sizes, expressed as the edema-corrected hemispheric lesion volume differed not statistically significant (5.18 ± 8.91% vs. 2.48 ± 3.65%, *p* = 0.931 *u*-test).

According to T2-weighted MR imaging after 24 h, no cerebral infarction could be detected in the control group (III). The infusion of air bubbles with a diameter of 45 μm (group I) and 160 μm (group II) resulted in ischemic infarction within the vascular territory supplied by the right internal carotid artery of four animals in each group (Figure 2 and 3). The assessment of infarct size showed a mean ischemic affected hemispheric volume of 5.18 ± 8.91% in group I and 2.48 ± 3.65% in group II, achieving no statistical significance (*p* = 0.931, Figure 3).

## Discussion

CPB-assisted surgical interventions remain one of the most important causes of CAM; thus, filter systems have been suggested to reduce the number of bubbles. Common filters are installed in the arterial line of extracorporeal circulation, acting as a strainer by trapping bubbles that are larger than its pores [19,20]. However, besides increasing resistance to the flow itself, progressive filling with debris and fibrin, activation of coagulation cascade, and the complement and immune response [7], it is possible that the structure of these filters causes dispersion of larger bubbles into numerous smaller ones. In consequence, this would increase the gas–liquid-endothelial interface, which is known to be a significant factor of CAM-mediated tissue damage by activation of inflammatory response, the complement as well as the clotting system [7-13].

Hence, it is reasonable to proof whether bubble size has an impact on the neurological outcome or the occurrence of cerebral infarction and if markedly expanded regions of ischemia may reflect the extensive increase of the gas–liquid-endothelium contact interface in turn.

For decades, technical problems hampered standardized and reliable investigation of cerebral air embolism in basic research such as problems of selective arterial injection, disruption of physiological blood circulation, excessive volume of the air embolus, and varying bubble size [21]. Most of these problems could be solved, but bubble sizes remained difficult to control: Nearly all animal studies on cerebral air embolism have used the injection of different volumes of air as a bolus or repetitively and titrated to a certain extent of damage without the opportunity to display and verify bubbles and their size [14,22-26].

To investigate the impact of bubble size, we used our recently established rat model for air microembolism allowing the application of gaseous bubbles under preserved physiological cerebral blood flow [15]. In the past, investigations on CAM revealed a dose-dependent toxicity of air volume and functional parameters: Gerriets et al. (2010) used a bubble diameter of 160 μm with an increasing number of bubbles, showing worse clinical outcomes at higher gas emboli counts [15]. Jungwirth et al. (2007) revealed a dose-dependent relationship between CAM and survival, neurological outcome, and histological outcome [24]. The present study compares two different bubble sizes yielding the same total injection volume of 86 nl, which -according to findings from preliminary experiments- seems to represent a vulnerable threshold for the reproducible provocation of cerebral infarction in a rat brain [15]. Technically, the custom-made device we used is able to produce bubbles with a diameter of 40-250 μm. Herein, we chose 45 μm for small bubbles and 160 μm for the larger ones, representing two different sizes within the range that our device can produce [15]. With respect to the number and diameter of gaseous bubbles, we could demonstrate feasible and highly reliable production and injection of small 45 μm-bubbles with a low standard deviation.

We were able to show that functional performance according to the Neuroscore worsened significantly after embolization when compared to the sham-operated rats. The tendency to larger bubbles leading to a worse functional outcome has to be interpreted with caution, because scores showed broad standard deviations and differences did not reach statistical significance here, which may be attributed to the low number of animals used. In our experiment, air microembolism regularly caused cerebral ischemic infarction for both bubble sizes, though differences in frequency of ischemia between group I and II could not be verified. Due to the extensive increase of bubble surface with smaller bubbles and the suspected noxiousness of the gas–liquid-endothelium contact, we considered ischemic infarction to increase in a comparable order of magnitude. The size of ischemic lesions, as detected by the MRI scan, appeared to be almost doubled for small bubbles; though, differences in lesion volume between the two groups did not reach statistical significance. In summary, we could not find any significant differences between the two bubble sizes concerning functional outcome parameters, number of infarctions, or lesion volume, despite a 45-fold increase in the number of bubbles, i.e. a 4-fold increase of total bubble surface respectively, in the 45 μm group.

Besides statistical caveats due to a reduced statistical power, further pathophysiological considerations may explain the absence of distinct effects: It seems to be reasonable but not proven that bubbles of different size will get stuck analogously in vessels of different sizes [14]. According to a dynamic bubble behavior, bubbles may not maintain their size and split, aggregate or dissolve during downstreaming within short time. This may lead to a uniform bubble size within the terminal vascular bed, causing comparable probability of infarction, lesion volume, and consecutive functional decline. Fur-thermore, there is experimental evidence that even gas bubbles small enough to pass through arterioles and the capillaries without verifiably occluding the vessel can lead to neurological deficits [22,27,28]. This may be supported by an observation from the venous system of the lung, where leukocyte and platelet accumulation around gaseous bubbles was observed on flowing bubbles [29].

The discrepancy between functional deterioration and infarct volumes in the intervention groups, supplemented by the observation that one rat in each intervention group showed clinical deterioration without displaying cerebral infarction on MRI, question cerebral ischemia as the only pathological correlate of CAM-mediated tissue injury. Clinical studies have revealed that not every patient with cognitive decline after CPB-assisted surgical intervention shows morphological changes such as ischemic stroke detectable by MRI, supposing that the majority of cerebral damages that become clinically obvious post-intervention is still beyond the detection level of MRI [2,30-33]. Not least because of the large number of different existing animal models, determinants and cut-off points for morphological or cognitive damage due to subtle CAM as well as pathophysiological changes with “substructural” damage are yet undefined and need more experimental data [7,24]. Our animal model of CAM offers a strongly controlled setting that may help to define thresholds to reproduce clinical deterioration without overt morphological changes in future.

Eliminating bigger bubbles seems to be insufficient to prevent post-intervention deterioration, and this may explain why supplements of currently used filter systems such as the dynamic bubble trap (DBT) lead to better clinical outcomes. Importantly, the DBT eliminates rather than fragments gas bubbles. Hence, clinical trials have shown a reduction of microembolic signals and CAM-associated effects with filter systems or a combination of filters with a bubble trap that reduced the number of bubbles [2,34].

It remains debatable, if the small effect size would have required higher animal numbers per group to increase statistical power in the end. As well, limitations of our study may include the number of dropouts, further reducing the number of animals completing the study protocol. There are no data revealing homogeneity of bubble sizes in the clinical setting or the relation between harmful bubble sizes and air volume in humans and animals. Additionally, the animals studied here were healthy, in contrast to multimorbid patients normally undergoing CPB-assisted surgery. Beyond that, susceptibility differences of species to ischemia have to be taken into account when findings are compared.

There is no denying that even the best animal model cannot simulate a clinical situation in detail. To concentrate on the plain cerebral bubble effect, we rejected interventions such as the heart-lung machine that may disturb the stability of vital signs by causing temperature changes or blood pressure dips, for example [24,35].

## Conclusions

Present methodological evaluation revealed the feasibility of standardized and controlled CAM, producing neurological deficits due to unilateral cerebral damage with comparably low mortality. The device used can reliably produce and inject even small gaseous bubbles with a diameter of 45 μm. In the context of a small study group, our findings could not display a strong dependency of stroke frequency and severity on bubble diameter. A trend towards larger ischemic infarction with small bubbles and worse functional outcome with larger bubbles did not reach statistical significance.

## Abbreviations

CAM: Cerebral air microembolization; CCA: Common carotid artery; CPB: Cardio-pulmonary-bypass; ECA: External carotid artery; %HLVec: Percent hemispheric lesion volume – corrected for edema; %HLVuc: Percent hemispheric lesion volume – not corrected for edema; HVc: Volume of the contralateral hemisphere; HVi: Volume of the ipsilateral hemisphere; ICA: Internal carotid artery; LV: Lesion volume; MRI: Magnetic resonance imaging; T2RT: T2-relaxion time; T2WI: T2-weighted imaging.

## Competing interests

The authors declare that they have no competing interests.

## Authors’ contributions

All authors read and approved the final manuscript. All authors listed have made substantive intellectual contributions to the study and this manuscript: MJ and MY participated equally in conception, rational, and design of the study, contributed in functional testing and drafted the manuscript. NS performed surgical interventions/ animal preparation, participated in application of microbubbles and functional testing as well as MRI-Imaging. JB made substantial contributions to concept, coordination and design of the study, accounted for cardio-surgical expertise, participated in statistical analysis. MN participated substantially in establishment of methodology and helped to draft the manuscript. MT partook in development and establishment of methodology, participated in MRI-Imaging, image-/ data-analyses (planimetry; assessment of infarct number and lesion volume) and statistical analysis. GB contributed fundamentally in MRI-Imaging, image-/ data-analyses (planimetry; assessment of infarct number and lesion volume) and administered radiological expertise. MK provided neurological expertise, helped to draft the manuscript and revising it critically for important intellectual content. PU contributed considerably to development, design and maintainance of the bubble generator, production of consumable parts of the device (i.e. capillaries, tubings etc.…) and generation and application of microbubbles during experiment. MS participated in development of bubble generator, made substantial contributions to the concept and design of the study, accounted for cardio-surgical expertise and funding. TG participated in development of bubble generator and establishment of methodology, conceived the study, participated in conception and coordination of the study, assured funding and helped to draft the manuscript.
